# Online Mental Health Forums and Rural Resilience: Mixed Methods Study and Logic Model

**DOI:** 10.2196/47459

**Published:** 2023-06-28

**Authors:** Artur Steiner, Jane Farmer, Peter Kamstra, Karen Carlisle, Anthony McCosker, Sue Kilpatrick

**Affiliations:** 1 Glasgow Caledonian University Yunus Centre Glasgow United Kingdom; 2 Swinburne University of Technology Centre for Social Impact Swinburne Melbourne Australia; 3 University of Melbourne Melbourne Australia; 4 James Cook University Townsville Australia; 5 University of Tasmania Hobart Australia

**Keywords:** online forums, personal resilience, mental health, rurality, logic model

## Abstract

**Background:**

Rural mental health is a growing area of concern internationally, and online mental health forums offer a potential response to addressing service gaps in rural communities.

**Objective:**

The objective of this study was to explore and identify pathways by which online peer support mental health forums help to build resilience for rural residents experiencing mental ill-health by contributing to overcoming their specific contextual challenges.

**Methods:**

We developed a Theoretical Resilience Framework and applied it to 3000 qualitative posts from 3 Australian online mental health forums and to data from 30 interviews with rural forum users.

**Results:**

Drawing on the findings and an abductive approach, a logic model was developed to illustrate links between the resilience resources built and enabling features of forums that make them spaces that facilitate resilience.

**Conclusions:**

The study demonstrated that online forums make valuable contributions to social well-being and access to a range of timely support services for rural people experiencing mental ill-health, and, while doing so, involve users in the processes of resilience building. The study provides a new way for practitioners to frame the work of and value produced by forums. It gives a logic model that can be used in evaluation and audit as it facilitates a causal framing of how forums, as an intervention, link with resilience outcomes. Ultimately, the study contributes to developing new knowledge about how rural resilience building can be conceptualized and measured while showing how forums are part of contemporary health service provision in rural places.

## Introduction

### Online Mental Health Peer Support Forums

Since the 2010s, online mental health forums have gained popularity as a way to give and receive information and emotional support between peers experiencing mental health conditions [1]. Factors influencing this trend include increased accessibility of the internet [2], evidence of the benefits of mental health peer support [3,4], and increasing provision of mental health and well-being services by nonprofit organizations focusing on holistic support [5]. Most recently, the COVID-19 pandemic accelerated public acceptability of web-based health services [6].

Studies provide evidence of multiple benefits from using online mental health peer support forums [7]. Forums offer timely access, are efficient in responding to multiple types of needs [8], and overcome geographical boundaries by linking people with similar interests [1]. Compared with the provision of traditional on-premises services, a unique advantage of online peer support forums is their ability to provide access to information from others with similar experiences who can provide advice in nonclinical language [9]. This creation of a space of relating and empathy formed by exchanges between forum users helps to develop trust [10,11], enabling people to discuss their needs and to develop strategies for coping within a safe, anonymous environment [12].

In this study, we were particularly interested in the aspect of developing coping abilities and *the use of positive coping mechanisms such as seeking social support, positive thinking and problem solving*—all associated with the concept of resilience [13]. Interestingly, although previous studies of online forum use show a range of benefits, including some that have looked specifically at rural users [1], we have found no study that specifically considered *how* online forums could help to build resilience for rural people experiencing mental ill-health. As such, we draw together existing discussions about online mental health forums and rural resilience, and in doing so, present new knowledge for the field. Next, we provide an explanation of resilience in the context of this study.

### Resilience in This Context

As a latent construct that is not objectively observable [14], resilience is conceptualized in different ways depending on the context. In social scenarios, it is suggested as a desirable collective resource for dealing with adversities, including responding to environmental disasters, lack of economic opportunities, inaccessibility of services, and public health challenges [15,16]. Community resilience has been discussed as building through proactive processes that continually add capacity for populations to respond to risks and effect change and involving persistence, adaptation, and transformation acting as interrelated phenomena allowing resilience to occur [17,18]. Resilience relates to the capacity to adapt and thrive by regarding “disturbances as an opportunity for change and development” [19]. It can be understood as both an ongoing process [20] and an outcome [21]. Magis [22] locates resilient community members in a relationship with resilient communities, suggesting a circular flow where resilient collectives help individuals by making resources available and where individuals then harness these resources to add to community capacity. She says resilience is “the existence, development, and engagement of community resources by community members to thrive in an environment characterized by change, uncertainty, unpredictability, and surprise. Members of resilient communities intentionally develop personal and collective capacity that they engage to respond to and influence the change, to sustain and renew the community, and to develop new trajectories for the communities’ future.”

A full description of the varying understandings of resilience is beyond the scope of this paper (for a detailed discussion of resilience in the rural context, refer to the paper by Skerratt [16]). Here, we understand resilience as having the individual resources to *adapt and evolve* to new circumstances after or while experiencing adversity [23] and *building strength* to deal with adversity and overcome vulnerabilities [20]. As we feature rural residents, we term this as “individual rural resilience.” However, reflecting back to the quote by Magis [22], findings are also relevant to other levels of resilience, involving understandings of collective or community resilience. This is because the collective of people using a forum can be understood as an *online community* where—by interacting on the web—forum users exchange resources for building resilience with each other. In addition, the people using the forums live in *geographically rural communities*, and thus, there is potential for positive gains in individuals’ resilience to contribute to collective resilience resources available to their geographical communities. This would happen by enabling people to participate in community life and by adding to the diversity of people who can contribute to community capacity [24].

Although governments and academics have proposed developing resilience as a way to respond to coping in challenging rural contexts, for individuals and communities, there has been less granular study of how specific interventions can influence rural resilience [17]. This results in a gap in tools to assist policy makers, capacity-building practitioners, and citizens to assess what is happening in resilience processes.

### Forums and Resilience Building

This study focused on rural people experiencing mental ill-health. It focuses on what happens *in* online mental health peer support forums, and it takes a resilience lens to understand the impacts that forums can have on users’ lives. Living in rural places is widely acknowledged to present specific challenges for people experiencing mental ill-health, including inaccessibility of health services, lack of confidentiality, and experiences of stigma and isolation [25]. Given the exacerbated experiences of adversity for rural people experiencing mental ill-health compared with those of urban dwellers [26], there is distinct value in understanding the mechanisms that build rural resilience and respond to their needs.

Building resilience for people with mental ill-health resonates with understandings of recovery for this group as about gaining resources to deal with a fluctuating condition. Duff [27] depicts a good outcome as achieving “health in illness” by navigating “an individual practice of health in perpetual tension with the enduring symptoms of mental illness.” Understanding how rural individuals experiencing mental ill-health can build resilience via online forum use is not only important in itself but also—by helping to unpack pathways to resilience—can help to inform ways to develop and assess other interventions targeting rural resilience [23,28].

Considering the rapid development of web-based services, this study aimed *to explore, systematize, and show a pathway by which specialized online peer support forums can act as an intervention to assist resilience building for rural people experiencing mental ill-health*. To do so, we developed a Theoretical Resilience Framework by using evidence combining rural community development and psychology perspectives. This draws on a chronology of multidisciplinary research by a team including Buikstra et al [20] and Berkes and Ross [23]. The framework (1) guided the analysis of post data generated by rural users of three online mental health forums hosted by Australian nonprofit organizations and (2) informed a topic schedule for interviews with rural forum users. In addition, by identifying resilience resources built, our data analysis reveals features of forums that make them spaces that enable resilience. By discovering *how* resilience builds and what enables it, we aimed to present a logic model that shows a pathway between using forums and rural resilience.

### Theoretical Resilience Framework

To address the study aims and assist with data collection and analysis, we used a narrative review approach [29] to inform the development of a Theoretical Resilience Framework (Table 1). A narrative review is a semisystematic literature review that is useful when analyzing complex, latent constructs, such as resilience [30], that are conceptualized diversely, making it challenging to conduct a fully systematic review [31]. Much of the literature about resilience takes a *rural community*–level perspective—that is, it considers collective population resources and structural resources (eg, economic opportunities, amenities, and services [32]). Another stream concerns *individual psychological* resources and is not contextually specific [33]. Context-free perspectives fail to acknowledge the layers of adversity experienced by rural people [26]. Our study targets the nexus of individuals experiencing *mental ill-health* and *living in rural contexts*. Few researchers target this specific area. Studies can be traced back to an initial multidisciplinary study of “shapers” of rural individual resilience [34]. Buikstra et al [20] refined and tested that work, offering a starting set of rural “resilience promoting factors.” Building from that study [20], we applied a snowball method [35] to identify other relevant literature. This involved (1) searching for later studies about resilience by authors of the original paper (ie, published after 2010) and (2) searching on Google Scholar for all recent papers that cited or were “related to” the study by Buikstra et al [20] (N=307 [cited or related]). From these studies, we included all those stating resilience factors that could relate to individuals (vs community-level factors; Table 1).

**Table 1:** Identifying individual resilience factors in the literature.

Studies with individual resilience factors	Resilience factors given	Individual resilience factors
Hegney et al [34]	The environment Connection with the place Family Culture Being part of a rural community and community spirit	Connection with the place Family Culture
Buikstra et al [20]	Social networks and support Positive outlook Learning Early experience Environment and lifestyle Sense of purpose Embracing differences Beliefs Leadership	Social networks and support Positive outlook Learning Early experience Environment and lifestyle Sense of purpose Embracing differences Beliefs Leadership
Berkes and Ross [23]	Social capital and networks Sense of place Values Social identity	Social capital and networks Sense of place Values Social identity
Maclean et al [36]	Knowledge, skills, and learning Community networks People-place connections Community infrastructure Engaged governance	Knowledge, skills, and learning
Leite et al [37]	Formal education Local knowledge Autonomy Family support Social capital and networks of support Access to psychological counseling An “invigorating environment”	Formal education Local knowledge Autonomy Family support Social capital and networks of support

Using this set of individual resilience factors, we applied an abductive approach to generate a “final” Theoretical Resilience Framework against which to analyze all data in our study. Abduction involves working between an initial general theory (in this case, the multiple resilience factors suggested in the literature) and specific data (in this case, forum posts) to arrive at a workable theory that accounts for empirical data [38]. To do this, 3 researchers analyzed a sample of 100 forum posts and found consistent evidence of 5 factors (shown in Table 2). On speculating why only these variables were repeatedly evidenced, we found that some other variables appear to be more descriptive of personal attributes that would be less changeable over time, for example, beliefs and early experiences. Other variables are challenging to identify in qualitative data sets—for example, sense of purpose, positive outlook, embracing difference, and leadership. Notably, the factors in the final framework all represent *resources that are subject to change*, which makes them useful for studying the building of resilience in response to an intervention.

**Table 2:** Identifying factors of the Theoretical Resilience Framework.

Initial rural individual resilience factors (from literature)	Retained or rejected based on sample analysis
Social capital and related social connections, social support, family support, and social networks	Retained
Sense of belonging (including connection to the place)	Retained
Learning knowledge, skills, and education	Retained
Self-efficacy agency, self-organizing, and autonomy	Retained
Adaptive capacity	Retained
Culture, values, social identity, and beliefs	Rejected
Positive outlook	Rejected
Early experiences	Rejected
Sense of purpose	Rejected
Embracing difference	Rejected
Leadership	Rejected
Environment and lifestyle	Rejected
Local knowledge	Rejected

Therefore, the final Theoretical Resilience Framework postulates that individual rural resilience, in the context of this study (involving people experiencing mental ill-health in a rural context), is characterized by (1) *social capital* or access to networks of people and the support, trust, and inclusion they can foster [20]; (2) *a sense* of *belonging,* which concerns acceptance as part of a group or community and the processes that lead to identity formation [16]; (3) access to *learning and knowledge,* including information about multiple topics, leading to increased capacity to navigate change [21]; (4) *self-efficacy* or being able to self-organize or work toward feeling in a controlled state [23]; and (5) *adaptive capacity* in the sense of having resources that enable adaptation and behavior change [39]. The framework was used to guide data collection and analysis as discussed in the following sections.

## Methods

### Overview

This study derives from a large project called o*ptimizing the roles of online communities in rural resilience* and funded by the Australian Research Council. To explore resilience processes, we obtained qualitative forum post data of rural users and interviewed rural forum users in relation to online peer support forums of 3 well-known Australian mental health nonprofit organizations—SANE Australia, Beyond Blue, and ReachOut. The organizations, and thus their forums, target specific demographic groups (Table 3). We analyzed the post data thematically using a Theoretical Resilience Framework (Table 2). To verify the resilience themes found in the post data and to understand more about the features that enable resilience, in-depth interviews were conducted with 30 rural forum users. Our literature review and the 2 data sets were used together to triangulate findings and to identify pathways between the use of online mental health forums and building resilience.

**Table 3:** Characteristics of host organizations, forums, and data collected.

	SANE Australia	Beyond Blue	ReachOut
Group targeted	People experiencing complex forms of mental ill-health including schizophrenia, bipolar disorder, and posttraumatic stress disorder	People experiencing anxiety, depression, grief, and posttraumatic stress disorder	Young people aged between 14 and 25 years (inclusive) and experiencing mental health issues
Goals of the forum	Advocacy, research, and support	Reduce stigma and discrimination and deliver better health outcomes for people in relation to anxiety, depression, and suicide	Prevent or delay the onset of mental health issues and reducing their incidence, severity, duration, and frequency
Posts from rural areas (N=193,356), n (%)	12,032 (6.22)	5027 (2.59)	11,905 (6.16)
Authors of posts from rural areas in the sample analyzed, n	251	684	121
Interviewees from rural areas (n=30), n (%)	20 (66.67)	6 (20)	4 (13.33)

In this study, we define rural areas as all areas outside of “Major cities” using the Australian Statistical Geography Standard (ASGS) Remoteness Structure [40], which categorizes locations based on populations’ relative accessibility to services. This is a measure of rurality that “makes sense” in an Australian context, as populations and services are concentrated in major cities. According to a recent review, 49% of studies used a measure of relative service accessibility to denote relative rurality [41].

### Ethics Approval

Ethics committee approval was granted by Swinburne University (R/2019/033).

### Participating Forums

Available nationally to Australians, the forums we studied are accessible to anyone with internet access. People must register to be a member, and this allows them to post and read others’ posts. We use the term “users” here to distinguish those who post compared with other members who do not post. Clearly, this is shorthand, and members might “use” the forums by reading and using information in posts while not posting themselves. Those who do not post were not included. Forum users can choose to post on existing threads or to create new topic threads. Forums are moderated by staff paid by the host nonprofit organizations. Moderation broadly involves ensuring that users do not disclose personal or identifiable information, removing spam content, removing discouraging comments, preventing prescriptive advice and abusive language, and diffusing conflict.

### Forum Post Data

To ethically collect forum post data, we ensured that our study aligned with the research principles of each of the host web-based mental health organizations [42]. We were able to use forum data because, when people register, they agree that their data may be used or reused for research purposes as long as it remains anonymous. Initially, a sample of deidentified forum posts (N=193,356 posts) was obtained, including from SANE (68,634/193,356, 35.49%), ReachOut (80,174/193,356, 41.46%), and Beyond Blue (44,548/193,356, 23.04%). Posts were collected from the period between August 2018 and December 2020. Data were “cleaned” before analysis to remove content that could be potentially identifiable, for example, web-based pseudonyms and references to named geographical locations, organizations, or people. Then, as we focused on rural residents, we selected all posts in which rural postcodes were provided when registering for membership. To obtain a sample that we could analyze in the time available, we further selected a sample of 1000 rural posts per forum (n=3000 posts). These 3000 posts included all posts from *very remote* (n=269, 8.97%) and *remote* (n=410, 13.67%) ASGS categories and a sample of 2321 (77.37%) *outer regional*–ASGS category posts randomized using the Excel (Microsoft Corporation) random function (ie, =RAND).

### Interview Data

A sample of rural user interviewees was recruited using a recruitment advertisement posted on each forum. The recruitment posts explained the study’s aims and invited rural users to contact the researchers using a web-based “expression of interest” form. The interviews with 30 rural forum users were conducted between 2021 and 2022. Owing to rural locations, COVID-19 restrictions, and respect for users’ anonymity, 80% (24/30) of the interviews was conducted via phone and 20% (6/30) was conducted via Zoom. Duration ranged from 45 to 60 minutes. A “duty of care” protocol was provided that offered support to interviewees to access counseling resources if required.

Interviews were semistructured and included questions about users’ experiences with using forums, the extent to which benefits or disadvantages accrue, and reasons for changes to occur. Questions about resilience themes identified in the Theoretical Resilience Framework were included. Interviews encouraged users to comment about other aspects of services and community life that influenced overcoming adversity, allowing for other information to emerge according to the interests, experiences, and views of the interviewees [35]. All interviews were audio recorded with consent and transcribed verbatim. Interviewees’ ages ranged between 18 to >65 years. Of the 30 participants, 11 (37%) identified as men and 19 (63%) as women. All Australian states were represented, but none of the participants were from Northern Territory or Australian Capital Territory.

### Data Analysis

Forum post and interview data were analyzed using a thematic qualitative analysis method [43]. First, data were analyzed deductively against the Theoretical Resilience Framework (refer to Table 4 for themes in data coded to resilience factors). Inductive analysis was also applied, to explore and discover (1) themes about challenges experienced by rural people and (2) themes about forum features that enable benefits for users. To increase the reliability of data analysis, 4 researchers (JF, SK, PK, and AM) initially read the data, noting themes independently. Agreement was then reached on an initial codebook outlining what would be included or excluded for each thematic code. Then, PK, AM, and KC systematically coded the forum posts and interview transcripts independently using NVivo (QSR International). These coded data were then discussed with the wide research team, and agreement was reached on any inconsistencies. At this point, the data were collected as rich bodies of posts and quotes for each theme. These were checked, and refinements were made regarding allocation according to theme by AS by rereading all coded material data. Agreement about inconsistencies was reached via discussion among the whole writing team. Some quotes or posts were allocated to >1 theme because content overlapped themes. In the findings, we have provided an overview of key insights along with illustrative forum posts (marked as Beyond Blue, ReachOut, and SANE) and interview quotes (using pseudonyms for anonymity).

**Table 4:** Data coded to resilience factors.

Resilience factor	Description of data coded to this resilience factor
Social capital	This category included descriptions about social connection, relatedness, or empathy between people, for example, expressions of friendship and friendly encounters such as peers offering encouragement to each other, referring to each other as friends, thanking peers for trusted friendships, and describing the value of these friendships.
Sense of belonging	This category included (1) people joining and posting messages seeking to belong to this community, for example, introducing themselves and telling their story; (2) posts that tell others that they belong; and (3) posts that reference the forum as a beneficial place, perhaps discussing features of the forum as if it is a physical place, and testimonials about forum benefits.
Learning and knowledge	This category included (1) giving knowledge, for example, strategies for coping with symptoms, including from lived experiences; (2) requests for advice or information, for example, how to access services; and (3) sharing web-based resources.
Self-efficacy	This category included descriptions relating to control or loss of control: (1) expressions relating to using the forum as a step to gain or regain control; (2) asking others on the forum to “hold them accountable”; and (3) where people tell their story to unload, sometimes with discussion suggesting that this helps to move on.
Adaptive capacity	This category included descriptions about how interacting on the forum has changed their activity, attitude, feeling, or knowledge.

### Developing a Logic Model

Logic models are descriptions of the chain of causes and effects leading from an intervention to an outcome or outcomes of interest, that is, in our case, individual rural resilience. Logic models identify, describe, and arrange critical aspects of an intervention to represent how the intervention produces change, with arrows used to indicate causal relationships between the aspects [44]. They are a useful tool, as they offer an explicit visual statement of the activities that will cause change and the results expected when implementing a specific intervention. As such, they assist in identifying essential project resources and support planning. They can inform the design of future interventions and policies [45,46].

To develop a logic model depicting a pathway by which forums help to build rural resilience, we adopted an abductive approach. As explained by Timmermans and Tavory [38], “theory construction” using abductive analysis requires a “dialectic between data and generalisation as a way to account for empirical findings.” That is, it involves a “back and forth” between data and theory to establish whether the data are a case of x. In our study, we focused on the patterns and links between the factors and enablers of resilience in the Theoretical Resilience Framework, the empirical data about these, and any evidence of causality.

## Results

### Overview

**Figure 1:**
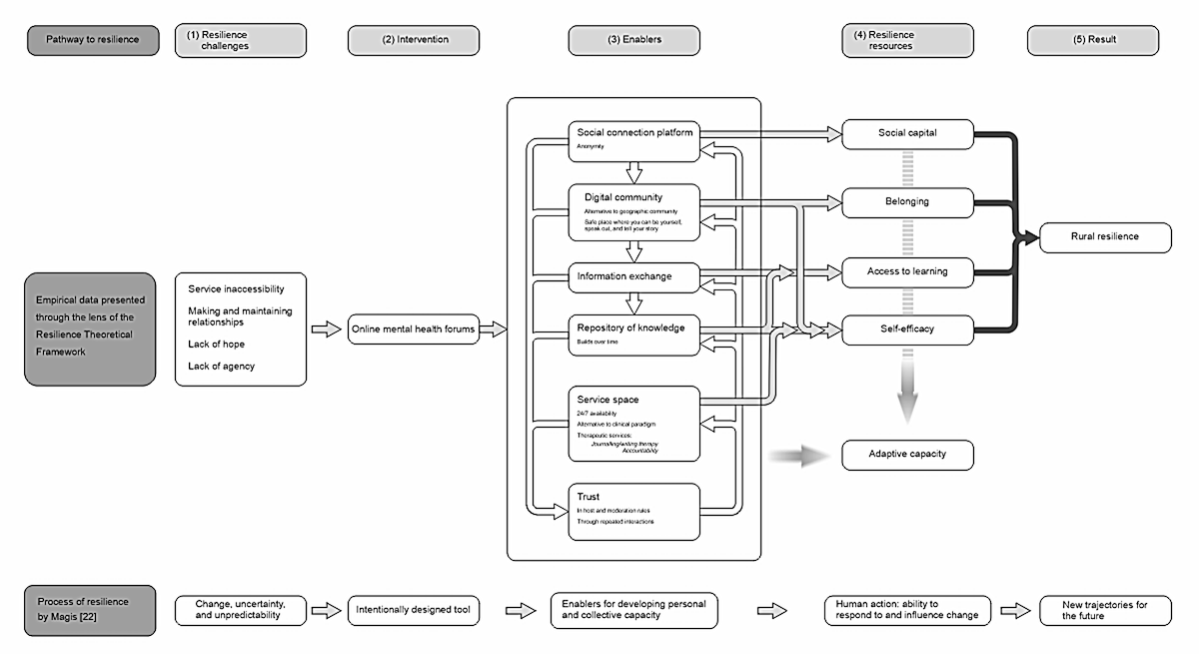
Pathways to rural resilience [22]. Please see the high-resolution version of this figure in Multimedia Appendix 1.

In this section, we first applied inductive analysis to forum post data to reveal the challenges faced by rural people experiencing mental ill-health that they report on forums. Second, we presented findings from deductive analysis applying the Theoretical Resilience Framework to show resilience resources from forum use. We used data from interviews to confirm that forum users acknowledge that they gain these resources. Third, by applying inductive analysis to interview data, we identified features of forums raised in interviews that show how forums enable resilience to build. Wherever we have provided forum posts as examples, the spelling and grammar has been kept exactly as in the posts. These are indicated by the name of the forum and the number of the post, for example, “Beyond Blue; post 1011.” In the *Discussion* section, we applied evidence from the *Results* section to develop a logic model that outlines a pathway showing how forums as an intervention can enable resilience resources to be built (Figure 1).

### Challenges Experienced

We grouped the challenges identified in the forum data into themes relating broadly to service inaccessibility, making and maintaining relationships, and lack of hope and agency. These are discussed in this section.

Users often paired mentions of their rural living with problems of *inaccessibility* to a spectrum of services including general practitioners, psychologists, counselors, mental health nurses, and psychiatrists. Challenges included nonexistence of some needed services, distance, lack of transport options, costs, and restricted choice—a crucial issue if the person had poor experiences with an available practitioner [47]. Illustrative posts include the following:

Today I drove 3 hours to the doctors to get my psych re-evaluation. I am falling apart. It’s official, I’m experiencing anxiety at unprecedented levels. To get there wasn’t easy.

Beyond Blue; post 1011

I am on the waiting list to DBT [Dialectical Behavior Therapy] but live in the middle of nowhere so that’s not an option right now.

SANE; post 3

How distance and inaccessibility exacerbated experiences of mental ill-health was frequently referenced; for example, a user described living on a cattle station 500 km from the closest town and struggling to cope with a new baby (Beyond Blue; post 3). Another resident of a small town referred to the debilitating impact on their agoraphobia, of trying to reach a specialist located >2 hours away (SANE; post 4).

Users discussed that relationships could be difficult to navigate in the context of isolation, with challenges in *making new relationships and navigating existing problematic relationships* affecting people’s mental health and ability to thrive and cope:

I have a few [friends] who live far away from me, but I am so lonely in the small town I moved to for work. I can’t talk to someone unless they talk to me first, and even then, I can’t look them in the eye.

Beyond Blue; post 27

When you live in a small village, everyone knows everything about everyone. You can’t date, you can’t go to a support group...because everything that you say will be repeated.

Beyond Blue; interview 3

Among the analyzed posts, there was a recurrent discussion about how to maintain relationships with friends and family while not “burdening” them with issues associated with users’ mental ill-health. These quotes illustrate that—at times—it is not isolation per se, rather concerns about the well-being of others, that prevents people from sharing their experiences and feelings:

My wife is my best friend and I do talk to her about it, I have mates but I don’t want to burden them with my issues.

Beyond Blue; post 21

I tried talking to a counsellor at school about it but she didn’t help. I can’t talk to my mother about it as she already has enough on her plate. I can’t tell my friends about it as I don’t want to burden them with my baggage. I just need someone to talk to.

Beyond Blue; post 823

Forum users talked about *lack of hope* and fears about how they would manage their condition in the future. They questioned what their life might be like going forward and expressed challenges with knowing what to do and how to achieve *the agency to make changes happen*:

Feeling like there’s just no point to your life. That happens to me a lot because I’m a carer to two special kids. And the prospects of my own life and my babies’ lives, I just wish that they were better but they’re not. It is what it is. And sometimes that gets on top of me.

SANE; interview 1

I am terrified that I will project my insecurities onto my children and cause damage to them in some way, and I am terrified that my issues will ruin my marriage or hurt my husband. I hate being this way, but I truly don’t know how to change.

Beyond Blue; post 93

Some of these challenges align with the findings of international studies and reviews about the experience of mental ill-health in rural areas. Studies tend to focus on inadequate mental health services [25,48] and experiences of stigma and lack of confidentiality [49]. An issue that is less often raised, but surfaces in forum data, is the personal emotional pain of trying to create satisfying relationships in isolated places and of the inescapability of negative relationships. Studies of lesbians, gay men [50], and transgender individuals in rural places confirm the challenges of “being different” [51]. Findings about feeling burdensome reflect more generic mental health experiences (ie, not confined to rural areas) [52], whereas lack of agency, despair, and hopelessness can be compounded in rural places where appearances must be maintained and there are few support groups [53]. This situation of social stigma and lack of places to relate and be meaningfully occupied could be linked to high rural suicide rates [54].

### Resilience Resources

By applying the Theoretical Resilience Framework, findings from the deductive analysis show how resilience resources are generated *for* and *by* users through their interactions on forums.

Building *social capital* can be seen through exchanges of posts in which people connect one-to-one, expressing support, caring, and identifying each other personally. These connections were made in specific threads of the forums, and we found evidence of ongoing conversation—from introductions to dialogues about personal feelings:

I’m glad things are starting to feel a little better for you and hope they continue that way. How is the assignment coming along? How amazing is it going to be when we’re all finished for the year?!

ReachOut; post 37

First, I want you to know, that you’re not alone in this world. It may not feel like it, but you have many people who love you, very very much- you just can’t see it. You are a wonderful person, with an amazing life– you raised children, had a long relationship.

Beyond Blue; post 100

Interviews confirmed that new social connections were made on the web. Although some of the connections were relatively abstract, as presented in the following quote, others turned into a form of friendship, with forum users expressing concerns about each other:

I’ve met people on the forums. And they will post something and tag me into the post. You know, I get a notification that someone’s mentioned me in a post. So, there’s that kind of camaraderie that’s built up.

SANE; interview 1

The idea of *belonging,* generated via engaging on the forums, was regularly expressed. Posts indicate that the value of being part of the forum as a community sometimes helped to overcome challenges of finding belonging in their geographical locale. Some users stated how, in a rural context, it is hard to find friends who are empathetic and prepared to challenge their own community identity to make people experiencing mental ill-health feel included. Users expressed relief when discovering that multiple people—on the web—understood what they were going through:

I don’t really have a support network. I live away from my family and have no friends. Because of this I can’t get out and meet people. But reading on here and knowing I’m not alone and I can come on here and talk to people who have or are going through the same as me has helped already.

Beyond Blue; post 400

Interviewees discussed how a sense of belonging is created on forums. This was often about existing users reaching out to new people to express understanding, support, and solidarity:

They weren’t telling me to ignore...things. It was more– “we know what you’re going through, we know how hard this is. We’ve been there.” “We’re here for you, talk as much as you want. Don’t worry about thinking what you’re saying is trivial. Or we think you’re just being stupid or that you're just being a pest for coming on here and saying how terrible you feel.” It was the support from people who’ve been there before.

Beyond Blue; interviewee 4

Interviewees referred to belonging being reinforced when they reached a point where they reciprocated support by giving help to others on forums. As such, our data show that belonging involves sufficient embedding and integration, that is, socialization [55], that a user is comfortable and confident to give and to receive assistance:

I find it helps me to be there and supporting other people. I tend to stick to areas where I know other people who’ve been on it, when they’re going through a rough patch to support them and vice versa. I try to support newbies, sometimes just by saying I’m listening...It’s more two ways now. I find it helps me being there for other people as well.

Beyond Blue; interview 4

Access to *learning and knowledge* is manifested in forum post data via direct and indirect requests for information and advice and users sharing information and advice in response or proactively. Information shared may include “factual” information about services, therapies, conditions, and experiences. Examples of such “factual” knowledge posts are as follows:

Another option is a Police and Ambulance Intervention Plan. This is a document that can be created to record what strategies would be useful for the police or ambulance officers attending to you in a mental health crisis.

SANE; post 117

Use aromatherapy and lavender is very calming. I have used it in a diffusor on my skin and on the palms of my hands rubbed together and inhaled it but never heard of the capsules. Would be interested to hear how they go.

Beyond Blue; post 237

I feel overwhelmed by small adult tasks and I am a huge stress head over almost everything. Please tell me it gets better? Or that I am at least not the only one?

SANE; post 75

Sometimes, users posted their viewpoints and opinions. Dialogues that involved series of questions and responses and reciprocal relationships in exchanging knowledge or advice were confirmed in interviews; for example, a forum user said the following:

Initially, I was using it, to seek help from other people, I guess, thinking that there might be someone who’s been through what I’ve been through. Over time, it became about giving help and information

Beyond Blue; interviewee 3

As such, the interview data prove the idea of a generated (online) community of interest of people who support each other by sharing information within an environment of support.

*Self-efficacy* was most clearly evidenced when users talked about gaining control or acknowledging the loss of control in aspects of their lives. Interacting in forums enabled people to express these feelings or self-assessments. In turn, this helped them to process their situation and how they got there and to set goals for themselves about where they want to be—in their future life or emotional state. Participants mentioned that the digital community helped them to “get things off their chest” and, in ways, to hold themselves accountable:

I was starting to lose hope that it can and will get better but I have to try remain positive that I can overcome this and give the medication time to fix the chemical imbalance as well as wait until I can see a mental health professional to learn coping strategies.

SANE; post 14

Being unable to speak about emotions with family, some people started using forums to cope with negative feelings as a way of taking control. For example, forums were frequently used for a form of journaling and as a platform for “letting things out” to ease off a negative state of mind:

Lost three important people this year. Living in a small Aboriginal community it’s hard to find someone who’s not a family member to just chat to. It’s hard to chat with family because they’re grieving too. I don’t know how to feel better anymore and it is starting to affect my home life and my work. I just wanted to be heard.

Beyond Blue; post 115

An interviewee explained how this element of being able to process things on the forum had become an important assistive tool:

It’s hard with that pressure to talk to someone because it’s difficult to articulate things, even in the moment. I need more time. So, I found that it was a better option to be able to sit there and take my time to write down things...So, as I’m writing I’m exploring, and I can do more, through the process of writing. I tend to come to a sort of light bulb moment in my understanding of myself.

Beyond Blue; interview 2

*Adaptive capacity* was coded when there was an indicated decision or change of activity, attitude, or mental state. In some posts, users discussed navigating life challenges and making decisions to help tackle the causes of these challenges:

Hi [username], thanks for ur [sic] reply. Yep, life seems to be picking up for me. Made a decision last week to leave the station [been here 3 yrs on my own]– going back to family farm.

Beyond Blue; post 79

Another aspect of adaptive capacity was expressed as awareness of personal issues and how to cope with them by acknowledging ongoing symptoms. This can include informing other users about strategies that worked:

I’m okay. I went away for two weeks with my family which kept me distracted and busy...it was kind of good to have a break but it can only last for a short time before I feel like I’m losing myself again, I get antsy and agitated and just need to get back to a space where I’m back on track with my discovery journey.

Beyond Blue; post 415

I was speaking to my doctor today about how much the conversation with you had changed my mood yesterday when things were very dark.

SANE; post 7

An interviewee explained how processes that they developed via their activity on the online forum had contributed to changing the way they interact with others in a physical setting:

Before I respond to something, I might re-read it two or three times or I might kind of read it, walk away and do something and then come back to it before I respond to it. Just so I can take that in. And that definitely spins off into when I’m actually physically speaking to people. I am a much better listener now than what I probably was previously.

SANE; interview 20

Therefore, consistent with those of other studies, findings show benefits for forum users from social support and access to knowledge [1]. We have been able to show that users’ engagement in forums contributes to addressing specific rural challenges and that the resources they build for themselves and others can be assessed as contributing to their rural resilience resources. Rural people are growing their social connections—giving access to *social capital*—and generating communities of people with shared experiences that help people feel *belonging*. Forums give users access to repositories of *learning* and enable them to gain control of their lives (*self-efficacy*). Using forums can lead to life changes, with *adaptive capacity* being key to resilience. In the following section, we discuss what it is about these forums that enables resilience to build.

### Resilience-Enabling Features

Interview data were useful for identifying features of forums that enable rural people to gain resources. These features mean that people use forums to meet their needs including obtaining assistance to solve problems. By being used to address peoples’ needs, we argue, forums can help to build resilience resources as shown previously. We now describe the enabling features discussed in user interviews.

*Anonymity* is described by users as making it easy to ask for advice and exchange information. Web-based anonymity may be particularly helpful in the rural context where people risk stigma if they “open up” to known people. Interviewees also suggested that avoiding in-person interactions can make it easy to start discussions and use them purposefully:

I’ve seen it’s mostly men that feel like they have to hide their true feelings in real life. And I guess, that’s just some subconscious programming from society. But with the anonymous forums, they can truly open up with what they truly feel as there’s no repercussions for them because no one knows who they are.

SANE; interview 12

Personally, for me, it [anonymity] really does [matter] because I don’t want somebody knowing the intricate details of what I might be going through, I like to try and pretend that I’m okay. So, I don’t want my name splashed everywhere. I do like being anonymous, but I also feel like that it can create those relationships between people.

ReachOut; interview 4

I’ve actually had anxiety myself, which I was diagnosed with three years ago. And in that time, I was looking all over the place for information and support. And I found online was more helpful for me because I didn’t know anybody.

ReachOut; interview 1

Providing an *alternative community* to their geographical community was noted as important by interviewees. This is depicted as a place where “people are not judging you” or “passing rumours around” (SANE; interviewee 7). An interviewee said the following:

I can go to my family when I’m ruminating or down about something or I’ve got a problem. But I don’t want to cross their boundaries either. Yeah, so that’s why it’s good to have kind of two communities that can support me there.

ReachOut; interviewee 2

The people in this alternative community are particularly valuable because they are empathic owing to having similar mental health experiences:

At many mental health services, you’re just scared of telling the truth because of how they might react to it. The people that are meant to help you...they just want to do stuff to you and control you. Other people [on the forum] have been through it. They get it. They just let you be who you are.

SANE; interview 7

Forums are valued as spaces where *you can be yourself, speakout, and tell your story* in a safe environment. Forums also facilitate *information exchange* by encouraging questions and people giving feedback; they facilitate customized responses and language that users understand. Forums were discussed as *repositories* that collect and organize specialized information that can be used at times of different needs:

I actually registered quite a long time ago...to view rather than write, to see other people’s experiences and to assist my understanding of my own condition. And to see how other people responded and get through their struggles.

SANE; interview 6

It’s a mixture of everything sort of coming together, because you might have one question, and then the next day have another question that’s kind of not related. But the answers you get, give you a way forward.

Beyond Blue; interview 5

Forums enable people to obtain help *at the times when they* *need it*, without having to wait to access on-premises services. An interviewee said the following:

It’s somewhere where I can kind of get help when I need it.

ReachOut; interview 1

Therapeutic benefits from using forums were also reported. Interviewees described writing on the forum as having a “journalling” function [56], where users can record things to unburden themselves in the moment and to help process difficult issues and emotions—as the following quotes highlight:

I found that it was a better option to be able to sit there and take my time to write down things. And that in itself is quite therapeutic, and also been widened to do it in a more targeted way. So that I’m on going to a thread that I relate to.

Beyond Blue; interview 2

It’s more that sort of short term– there’s something that’s really bothering me at the time, and I can’t move past it. [Using the forum] helps get me over that. And then I can bring back in the CBT that I’ve been doing. And kind of work from there. But it’s just getting over that initial hurdle. Sometimes there’s something that's bothering me and I can’t move past it until I’ve just spoken it out with someone.

ReachOut; interview 3

Interviewees used the forum to post about goals or changes, thus creating a level of *accountability* for personal actions:

Being part of the forum made me more accountable to myself...I was going to do this course, blah, blah. And then I had a bit of a think about it while they were talking and realised that I’d been telling myself that for seven years...Anyway, recently, I’ve managed to enrol in it. And it’s actually happening.

SANE; interview 19

Interactions on forums are experienced as authentic, with responses from “real people”:

I have found that really good. It’s a bit more personal. I do feel like I’ve had an interaction afterwards.

ReachOut; interviewee 2

Interviewees described how forums work as *an alternative to the clinical paradigm* because “people are so incredibly open, it’s like looking into their life...it’s written so personally. And everything else that I had been reading was so clinical” (SANE; interview 8).

Regarding the functional benefits from forums, there was repeated discussion about *trust*. This comes from trust in the reputation of the organizations that run forum [57] and trust that is formed through users’ ongoing anonymous exchanges. Being on the forums is experienced as a space of consistent, clear rules:

I actually trust the forum community more than I trust my local community...there are very few services for mental health here and like the psychiatrist comes once every couple of months so the forum is really important in between times...it’s just that I’ve built that trust over time. Because they’ve been there for me for so long.

Beyond Blue; interview 6

This space overall is run by professionals, and they have people that are qualified to deal with everything. So, you know that even if the people on the forum are making you feel a certain way, then the professionals are going to like step in and help you with it.

ReachOut; interview 2

As these comments show, interviewees identified forum features that make these web-based spaces particularly useful for meeting their needs as people with experiences of mental ill-health living in rural contexts. We contend that forum features are found to be so helpful that people use the forum for benefits and, *in doing so, build resilience resources*. The forum’s features enable users to build “alternative communities” that give safety and connection, and forums facilitate access to useful services that fill gaps and act to complement their mental health service system experiences. In both community and service dimensions, the continuing forum activity is fueled by trust from user interactions within a web-based environment supported and moderated by forum hosts.

Finally, we looked for negative features, but few were raised. Some users said they could not get the specialized information they need (eg, relating to a rare condition). Others commented about challenges arising from moderation practices, such as not being able to specifically reference risks or express anger and, sometimes, when people were banned or suspended, they simply “disappeared” from the forum, causing concern for other users who did not know where they had gone.

## Discussion

### Principal Findings

This study aimed to explore, identify, and systematize a *pathway* by which moderated online peer support mental health forums can act as an intervention to enable resilience building for rural people experiencing mental ill-health. We found consistent evidence suggesting that users gain access to *resilience resources* from using forums, according to a Theoretical Framework of Resilience that we applied. From interviews, we identified features of forums that enabled people to meet their needs. We understand these features as enablers for building resilience resources through using and interacting on forums.

To illustrate a pathway to resilience, we developed a logic model (Figure 1). A logic model simplifies complex situations, but this “paring down” assists to understand processes associated with specific interventions, while also generating a theory for further testing. Having a way to convey the resilience resources built via an intervention and features that enable these “on a page” is useful for policy makers and practitioners to communicate how initiatives work to effect change (eg, to funders, wide groups of politicians, or communities). Having a model is also useful in highlighting the stages where, in processes of change, assessment could occur. Such assessments could take the form of measurements or checklists (eg, do we see that x, y, and z features are in place because having these should lead to resilience building). Defining stages in this way means applying a logic model that can be useful to guide evaluation or for audit. In our study, via the data sources we used, we also indicate that, increasingly, evaluators can access more diverse sources of data to understand what is happening in complex social processes [42,58]. Here, we used forum post data to show and substantiate the generated resilience resources and a more traditional data source—interviews—to confirm findings and explain causal processes.

### Explaining the Logic Model

#### Overview

The logic model (Figure 1) draws on the findings to show (1) consistent challenges reported by rural forum users; (2) the forum as intervention; and (3) how the forum supports resilience building via its enabling features, with activity reinforced via trust that both builds from ongoing forum interactions and enables future interactions. In the logic model, we grouped the enablers to highlight how they act to formulate types of “enabling spaces” in the forum: a social connection platform, a digital community, an information exchange platform, and a repository of knowledge—with each of these spaces and its features feeding into and supporting the delivery of the next (shown by downward arrows). The forum also provides a space giving access to a range of services. In our conceptualization, the way the forum works to support benefits aligns with the discussion by Duff [27] that mental health coping and recovery are assisted by composite factors and cannot be dependent only on an individual and their response to their condition. Here, the composite factors include the user, other forum users, host organizations, and features enabled by the interplay between people and aspects of the forum technology. In his study, Duff [27] discusses how people experiencing mental ill-health can find alternative spaces where they feel safe. He describes different physical places and the assemblages they comprise. In our study, we are able to show a web-based space that appears to work in supportive and therapeutic ways for rural people who cannot access a physical place of refuge.

#### Resilience Resources Built

#### Overview

In this section we show links between enablers and 4 of the 5 resilience resources built. The links with “adaptive capacity” seem less clear. We found evidence of adaptive capacity, but perhaps, it is an emergent feature. Adaptive capacity manifested differently in the forum data (or at least we coded it as a different type of factor) because it is evidenced through data indicating objective change. Adaptive capacity may be a resilience resource or a resilience outcome. Some framings of resilience suggest that adaptive capacity *is* resilience [21,23], whereas others suggest adaptive capacity as a “a latent property, which can be activated when people exercise their agency” [23], suggesting that it is associated with activating self-efficacy. Social learning is also associated with adaptive capacity [59], but learning itself may be antecedent to self-efficacy. Berkes and Ross [23] note that there is a gap in literature explaining how resilience factors relate to each other. Here, we are limited to saying that the resilience factors were observed, but there is inconclusive evidence that some are antecedent to others (note: the logic model does not sequentially follow the sections in the *Results* section because, in analysis, it was first necessary to establish the existence of resilience factors, before identifying enabling features).

To illustrate how the logic model relates to data, we selected themes in findings to explain a pathway to resilience for an imaginary forum user—Jack.

#### Challenges

Jack has a new diagnosis and is struggling to access information and services in their isolated community. They are losing hope and do not know how to take control.

#### Intervention

Using a mental health peer support forum is suggested by their mental health nurse.

#### Enablers

Jack registers and, at first, they observe what other users are saying and perform some searches of the forum threads for topics relevant to their condition. Over time, Jack gains confidence to ask questions. They make friends with users, Happy123 and Hope678, who are supportive, and Jack is able to ask them how they cope and use Jack’s own language. After some time, Jack tells them about their own health journey. It is very personal, but the forum is anonymous; therefore, people will never know who Jack really is, and no one in their geographical community will know about these web-based interactions. The forum is a safe place because if anyone posts abusively on the forum, the posts are filtered out. Happy123 and Hope678 are friendly, but they also give Jack coping strategies and support control taking. Jack starts to give themself small challenges such as finding local exercise classes and starting to speak with neighbors. Jack starts to use the forum less for their own needs, but they know it is always there for Jack if their world goes bleak and they need immediate help. Jack also uses the forum to see if they can help and support others. These exchanges make Jack feel as a valued part of a reciprocal community.

#### Resilience Resources

Jack’s access to knowledge and learning, a social network, and belonging to an alternative digital community of shared experience have helped them to gain control. Access to these resilience resources has stimulated Jack’s innate capability to adapt.

#### Outcome

In our terms, we can say that Jack is building their individual rural resilience.

The final line of the logic model compares what has been observed and systematized from the data with the description of resilience by Magis [22]. In a phrase, Magis [22] comments simultaneously about individual resilience, community resilience, and the relationship between individual and community resilience. In relation to our study, we observed what Magis [22] refers to “the existence, development, and engagement of community resources by community members to thrive in an environment characterized by change, uncertainty, unpredictability, and surprise.” Magis [22] says that members of resilient communities “intentionally” develop capacity, and it *could* be said that people use the forums with intent—intending initially to help themselves and then to help others. Our findings also resonate with those in the study by Magis [22] in that we see people engaging to develop *personal and collective capacity.* People initially engage to help themselves, but they build a collective, ongoing resource that replenishes, refreshes, and changes—potentially to meet the needs of multiple users. Through our study, we see rural people appreciate forum resources to address challenges of their context. In its organicism and malleability, it is possible to suggest that the forum as a web-based community is resilient; however, for its ongoing existence, it is dependent on external enabling structures—that is, the host organization, its moderation practices, and its ability to generate ongoing funding to enable its continuation [60]. This points perhaps to the difference between a collective of resilient individuals making up a resilient community and the idea that a resilient community requires more than resilient individuals—it also requires high-quality supportive structures and governance [61].

### Implications for Academia

Findings of this study suggest that resilience is a composite resource or outcome that can be built through using a web-based intervention if the right conditions (enablers) are in place. Although previous studies show the benefits of online mental health forums [1,3,11], only a small number of them are specifically about rural contexts, and they do not look specifically at *how* online forums relate to resilience. In addition, by establishing resilience resources from online forums for rural people, our paper shows a causal pathway from an intervention to resilience. In doing so, it provides a theory for further testing—relating to *how* resilience can build through types of interventions, in specific contexts and for key groups.

### Implications for Policy

The logic model assists in understanding what happens in the processes of resilience building. The logic model is useful for policy, as it summarizes the features of an intervention that needs to be in place to build resilience. It deconstructs the features of the studied intervention and suggests how these lead to the realization of resilience resources. Knowledge gained from this study could inform the creation of tools to support the development of other resilience-building interventions and to assess their impacts. The logic model is useful for policy makers whose time and budgets are limited, as it can be used as an evaluative framework to guide the measurement of the effects of forums as interventions.

The study shows that resilience can be built through an intentional intervention that is web-based and, thus, relatively cost-effective. Further testing of forums and other types of digital platforms as enablers of resilience generation is required. As in our study, such initiatives complement on-premises services and should not be viewed as a replacement for rural services, as we have highlighted the limits of the services they can provide. Mental health peer support forums are useful in providing 24/7 access and some therapeutic services and should be considered as a part of rural health service systems when these are examined. Importantly, our findings suggest online forums as a preventive service readily on hand to help rural residents to cope with daily struggles, support their well-being, and help to build resilience—all contributing to easing the pressure on clinical service infrastructure.

### Implications for Practice

Resilience from using online forums can be regarded as a bottom-up phenomenon generated through the labor of many forum users and nonprofit organization staff, harnessing interactions and applying rules about conduct and communication. Through forum interactions, users harness connections and knowledge and play an active role in decision-making and in their own processes of health and recovery. Although having impacts on other users, they influence the course of their own life. Considering this intertwined relationship between forum users, the logic model helps to make sense of the processual nature of developing resilience, its feedback loops, and the interconnections between enabling features and resilience resources. The logic model systematizes factors and resources and their relatedness, identifying and describing critical aspects and showing how—in the case of our study—online forums help to enable changes for people. The abstracted but evidence-based logic model helps to highlight how the collective effects of forum interactions support change for individuals and, potentially, at the community level. This idea of stepping back to see the big picture of how interventions effect change is significant for practitioners in helping them to understand the outcomes of day-to-day practices.

### Strengths, Limitations, and Future Studies

Findings result from analyzing data relating to 3 online forums and from 2 types of data—interviews and forum posts. The triangulation enabled verification of the findings and understanding of causality. On the basis of analysis of large data sets, our study suggests consistent findings with respect to resilience resources. In saying this, we limited our analysis to using 1 framing of resilience. Coding was conducted according to our interpretation of the resilience theory and the framework developed for this study. There are other interpretations and frameworks of resilience.

Forum users are anonymous; therefore, we cannot comment about their sociodemographic profile. Interviewees were a mix of ages and self-reported gender and from a mix of locations across Australia. There were more interviewees who used SANE forums compared with the others, potentially because SANE interviewees were sought during the COVID-19 lockdowns, whereas others were sought after the lockdowns ceased.

Although there are indications that forums help people to engage more in their geographical community, we are unable to say that they transfer their resilience resources to make their geographical community more resilient. So—in turn—although people on forums may “develop new trajectories” [22] for their and the web-based communities’ future, we cannot comment about the impact of the forum on the future of the geographical community*.*

Findings relate to resilience for a specific group of people in context; therefore, they are not transferable to other peer support forums and contexts. Future studies might examine the transferability of resilience built via mental health forums, for dealing with emergent challenges, for example, disaster preparedness.

### Conclusions

The study aimed to explore, systematize, and show a pathway by which specialized online peer support forums can act as an intervention to assist resilience building for rural people experiencing mental ill-health.

By developing a new, evidence-based Theoretical Resilience Framework, we show the resilience resources built. Furthermore, via the mixed methods deployed, we reveal the features of forums that enable them to be contextually suitable. From this, we can see that, in using forums, the rural user group built resilience resources as a by-product of meeting their most immediate needs.

We used evidence to inform a logic model showing a pathway from forum as intervention to resilience resources built via the forum’s enabling features. This illuminates new understanding of the causal mechanisms of forums that can be used by forum host organizations and policy and can inform those developing rural mental health systems about the role of forums and about the gaps in formal public services they appear to fill.

Forums work by harnessing users’ collective labor, enabled by organizations that are attuned to peoples’ needs but also operate according to a set of clear guidelines and practices, enabling trust. There are lessons to be learned about the roles that web-based services can play in contemporary health systems and more widely about how health organizations can operate to facilitate services in responsive partnerships with users, rather than simply providing services according to outdated formulas.

## Appendix

Multimedia Appendix 1 High-resolution version of Figure 1.

## Abbreviations

ASGS: Australian Statistical Geography Standard
